# P-1530. Evaluation of Phenotypic Cross-Resistance between Cefiderocol and β-lactam/β-lactamase inhibitor combinations against *Pseudomonas aeruginosa* isolates from US Medical Centers

**DOI:** 10.1093/ofid/ofae631.1698

**Published:** 2025-01-29

**Authors:** Sean T Nguyen, Boudewijn L DeJonge, Jason J Bryowsky, Joshua Maher, Rodrigo Mendes, Miki Takemura, Yoshinori Yamano

**Affiliations:** Shionogi Inc., Florham Park, New Jersey; Shionogi Inc., Florham Park, New Jersey; Shionogi Inc., Florham Park, New Jersey; JMI Labs, Liberty City, Iowa; JMI Labs, Liberty City, Iowa; Shionogi & Co., Ltd, Toyonaka, Osaka, Japan; Shionogi & Co., Ltd., Toyonaka, Osaka, Japan

## Abstract

**Background:**

Prevalence of multi-drug resistant *P. aeruginosa* isolates has been increasing with cross-resistance reported among β-lactam/β-lactamase inhibitor (BL/BLI) combinations. This study evaluates cross-resistance between anti-pseudomonal BL/BLI combinations and cefiderocol (CFDC) against various non-susceptible (NS) subsets of *P. aeruginosa* isolates collected from US hospitals participating in the SENTRY surveillance program.

Susceptibility of cefiderocol (CFDC), ceftazidime/avibactam (CZA), ceftolozane/tazobactam (C/T), and imipenem/relebactam (I/R) against various non-susceptible subsets of P. aeruginosa isolates from US hospitals participating in the SENTRY surveillance program during 2020-2022

**Figure:**
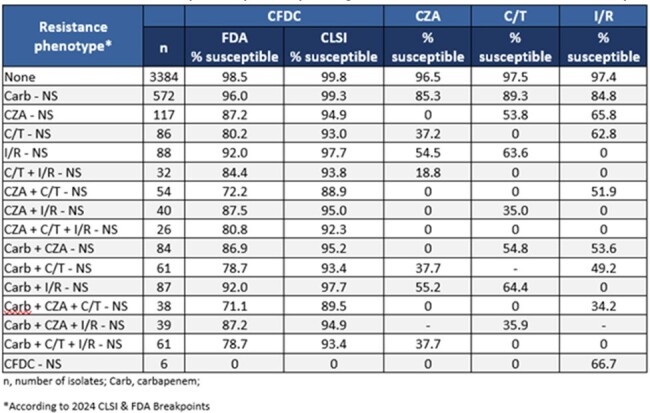

**Methods:**

A total of 3,384 clinical *P. aeruginosa* isolates were collected between 2020-2022 from hospitalized patients in 34 US hospitals as part of the SENTRY Antimicrobial Surveillance Program. Minimum inhibitory concentrations were determined according to CLSI guidelines using broth microdilution with cation-adjusted Mueller-Hinton broth (CAMHB) for comparator agents and iron-depleted CAMHB for CFDC. Susceptibility was assessed according to 2024 CLSI and FDA breakpoints. Carbapenem-non-susceptible (CarbNS) was defined as non-susceptibility to meropenem and imipenem.

**Results:**

Cross-resistance was observed among ceftazidime/avibactam (CZA), ceftolozane/tazobactam (C/T), and imipenem/relebactam (I/R). Of the C/T-NS *P. aeruginosa* isolates, only 37.2% of isolates and 62.8% of isolates were susceptible to CZA and I/R, respectively. In CZA-NS isolates, 53.8 % and 65.8 % tested as susceptible for C/T and I/R. Among I/R-NS *P. aeruginosa* isolates, 54.5 % and 63.6 % tested as susceptible for CZA and C/T, respectively. BL/BLI cross-resistance does not appear to affect susceptibility to CFDC, as >80% and ≥93% of CZA-NS, C/T-NS, and I/R-NS isolates tested as susceptible according to FDA and CLSI breakpoints, respectively. In various BL/BLI-NS ± CarbNS phenotypes, CFDC susceptibility was >88 % (CLSI breakpoints) while the various BL/BLI combinations susceptibilities ranged from 0-64.4 %. CFDC-NS was rare (< 1%), and none of these isolates were susceptible to CZA or C/T.

**Conclusion:**

In clinical isolates derived from US hospitalized patients, CFDC was highly active against *P. aeruginosa* NS isolates to BL/BLI combinations, whereas cross-resistance was observed amongst these combinations. The data supports the use of CFDC as an important treatment option against *P. aeruginosa* NS to BL/BLI combinations.

**Disclosures:**

**Sean T. Nguyen, PharmD**, Shionogi Inc.: Employee **Boudewijn L. DeJonge, PhD**, Shionogi Inc.: Employee **Jason J. Bryowsky, PharmD, MS**, Shionogi: Employee **Rodrigo Mendes, PhD**, Shionogi & Co., Ltd.: Grant/Research Support **Miki Takemura, n/a**, Shionogi & Co., Ltd.: Employee **Yoshinori Yamano, PhD**, Shionogi & Co., Ltd.: Employee

